# Efficacy and safety of intensity-modulated radiotherapy alone versus intensity-modulated radiotherapy plus chemotherapy for treatment of intermediate-risk nasopharyngeal carcinoma

**DOI:** 10.1186/s13014-020-01508-4

**Published:** 2020-03-16

**Authors:** Omer Aftab, Shufang Liao, Rongjun Zhang, Nan Tang, Meiqing Luo, Bin Zhang, Sanjeev Shahi, Raju Rai, Jazib Ali, Wei Jiang

**Affiliations:** 1 Department of Radiation Oncology, Affiliated Hospital of Guilin Medical University, 15 Lequn Road, Guilin, 541001 People’s Republic of China; 2 College of International Education of Guilin Medical University, Guilin, 541001 PR China; 3 Department of Medicine, Nanxishan Hospital of Guangxi Zhuang Autonomous Region, Guilin, 541004 China; 4 Department of Medical Oncology, Affiliated Hospital of Guilin Medical University, Guilin, 541001 China; 5 Department of Radiation Oncology, Wuzhou Red Cross Hospital, Wuzhou, 543002 China; 6 Department of Oncology, People’s Hospital of Gongcheng Yao Autonomous County, Guilin, 542500 China

**Keywords:** Intermediate risk, Intensity-modulated radiation therapy, Chemoradiotherapy, Nasopharyngeal carcinoma

## Abstract

**Background:**

This study directs to evaluate the efficacy and safety of intensity-modulated radiotherapy (IMRT) alone versus IMRT plus chemotherapy in intermediate-risk NPC (stage II and T_3_N_0_M_0_).

**Methods:**

A total of 124 patients with stage II and T_3_N_0_M_0_ NPC were pair-matched (1:1 ratio) to form two groups: an IMRT-alone group and an IMRT/chemotherapy group. Survival outcomes (overall survival [OS], disease–free survival [DFS], locoregional relapse–free survival [LRRFS], distant metastasis–free survival [DMFS]) and treatment-related grade 3–4 acute toxicity events were compared between the groups.

**Results:**

Survival outcomes for patients with stage II and T_3_N_0_M_0_ NPC were quiet comparable between patients treated with IMRT alone versus patients treated with IMRT/chemotherapy: 5-year OS was 91.9% vs. 90.3%, respectively (*P* = 0.727); DFS was 87.1% vs. 88.7%, respectively (*P* = 0.821); LRFFS was 96.8% vs. 95.2%, respectively (*P* = 0.646), and DMFS was 91.9% vs. 91.5%, respectively (*P* = 0.955). Grade 3 acute toxicities were significantly higher with IMRT/chemotherapy than with IMRT alone: mucositis, 15% vs. 5% (*P* = 0.004); leukopenia/neutropenia, 8% vs. 1% (*P* <  0.015); and nausea/vomiting, 22% vs. 3% (*P* <  0.001).

**Conclusion:**

For intermediate-risk (stage II and T_3_N_0_M_0_) NPC patients, the addition of chemotherapy to IMRT does not appear to provide any survival benefit. Moreover, grade 3 acute toxicities are also more common in patients receiving IMRT plus chemotherapy.

## Background

Nasopharyngeal carcinoma (NPC) is common in the southern regions of China, particularly in Guangdong and Guangxi provinces [1, 2]. Because of the biological characteristics of NPC, radiotherapy is the primary treatment modality.

Earlier, in the era of two-dimensional radiotherapy (2D-RT), chemoradiotherapy was the standard treatment for intermediate-risk NPC (stage II and T3N0M0). Concurrent chemoradiotherapy (CCRT) was shown to provide a considerable survival benefit for patients with stage II NPC [3, 4]. At present, “three-dimensional conformal radiotherapy (3DCRT) and the more advanced intensity-modulated radiotherapy (IMRT) have largely replaced 2D-RT, which enable the delivery of a higher and more accurate dose to a tumor target while sparing organs at risk [5], but CCRT is still considered the most suitable treatment for locoregionally advanced NPC [6]. However, some researchers reported that patients treated with CCRT have similar survival outcomes as those treated with only IMRT [7]. Moreover, stratified analysis demonstrated that, the addition of chemotherapy to IMRT did not significantly improve survival in stage II NPC subgroups. For example, Chen et al. and Sun et al. reported that the stage T3N0M0 subgroup have similar survival to stage II [5, 8] . Whether CCRT is superior to the IMRT alone for intermediate-risk NPC needs to be clarified. Hence, we included stage II and T3N0M0 disease as intermediate-risk NPC in our study, which would help in establishing individualized IMRT treatment protocols for stage II and T3N0M0 NPC.

The focus of the present study was to compare the efficacy and safety of IMRT alone versus IMRT plus chemotherapy in intermediate-risk (stage II and T3N0M0) NPC patients.

## Materials and methods

### Patients

The patients for this retrospective study were selected from among those hospitalized between 2011 and 2014 in the Department of Radiation Oncology at the affiliated hospital of Guilin Medical University, Nanxishan Hospital of Guangxi Zhuang Autonomous Region and the Wuzhou Red Cross Hospital. Patients were eligible for inclusion in this study if they had 1) newly diagnosed, intermediate-risk (stage II and T3N0M0) NPC; 2) Eastern Cooperative Oncology Group performance status ≤1; and 3) completed radical IMRT with or without chemotherapy (i.e., induction chemotherapy, neoadjuvant chemotherapy, and/or CCRT). Patients were excluded if they had 1) history of previous anticancer treatment or 2) history of another malignant tumor.

Patients were pair-matched [9] to maximize comparability between groups. Matching was performed for the following factors, with a descending hierarchy of priority: treatment regimen (IMRT vs. IMRT–chemotherapy); T category (T1 vs. T2 vs. T3); N category (N0 vs. N1); TNM stage (II vs. III [T3N0M0]); sex (male vs. female); age (≤ 45 years vs. > 45 years); and WHO histology (type II vs. type III). When multiple matched-pair combinations were possible, the pair with the closest admission dates was selected. All pairs were matched for at least five of the seven factors. Thus, we had two matched groups: one group comprising patients treated with IMRT alone (the IMRT-alone group) and another group comprising patients treated with IMRT plus chemotherapy (the IMRT/chemotherapy group). Survival outcomes and treatment-related toxicities were compared between the groups.

This study was approved by the Medical Ethics Committee of Affiliated Hospital of Guilin Medical University, Nanxishan Hospital of Guangxi Zhuang Autonomous Region and Wuzhou Red Cross Hospital. The need for informed consent was waived in view of the retrospective nature of the study.

### Pretreatment workup

Initial workup included clinical and laboratory examinations (hematologic and biochemistry profiles); fiberoptic endoscopy of nasopharynx; magnetic resonance imaging (MRI) or contrast-enhanced computed tomography (CECT) of the head and neck to evaluate the extent of the primary tumor and regional lymph nodes involvement; and bone scintigraphy, chest radiography or CECT, and ultrasonography of the abdominal region to exclude distant metastasis. All patients were restaged according to the 7th edition of the Union International Centre le Cancer /American Joint Committee on Cancer (UICC/AJCC) system [10].

### Radiotherapy

All patients received radical IMRT with 6-MV X-rays. The gross tumor volume (GTVnx) included the primary tumor as defined on MRI. Metastatic cervical lymph nodes were defined as GTVnd. The high-risk region was defined as clinical target volume 1 (CTV1) and included the whole nasopharyngeal cavity plus the GTVnx, with a margin of 5–10 mm. The low-risk area was defined as CTV2 and encompassed CTV1 with a margin of 3–5 mm, clivus, parapharyngeal space, skull base, sphenoid sinus, pterygoid fossae and the lower neck, cervical lymph nodes and the supraclavicular lymphatic drainage region have been included in CTV2 according to the NCCN guideline. The total prescribed dose was 66–70 Gy/31–33 fractions to the planning target volume (PTV) of GTVnx and 66–70 Gy/32–33 fractions to the PTV of GTVnd; a dose of 56–60 Gy/30 fractions to the PTV of CTV1 and 50 Gy/30 fractions to the PTV of CTV2 were delivered with first 30 fractions. All patients received one fraction per day, 5 days per week.

### Chemotherapy

Patients in the IMRT/chemotherapy group received three cycles of chemotherapy (cisplatium, 80 mg/m^2^) concurrently with radiotherapy, with intervals of 21 days between cycles.

### Follow-up

Follow-up duration was calculated from the first day of therapy to the date of last examination or death. At each follow-up visit, patients underwent physical examination, nasopharyngoscopy, ultrasonography of the abdomen, and chest radiography. CT scan or MRI of the head and neck region was conducted every 3 months during the first 2 years, and then every 6–12 months until the end of the study or death. For patients with suspected locoregional recurrence or distant metastasis, additional examinations were performed at the discretion of the treating physician. The primary endpoint was overall survival (OS), and the secondary endpoints were disease-free survival (DFS), locoregional relapse–free survival (LRRFS), distant metastasis–free survival (DMFS), and treatment-related toxicity. OS was defined as the time from registration to death from any cause or last follow-up; DFS was defined as the time from registration to treatment failure or death from any case; LRRFS was defined as the time from registration to first local or regional relapse or last follow-up; and DMFS was defined as the time from registration to first detection of distant metastasis or death from any cause. The Radiation Therapy Oncology Group (RTOG) radiation morbidity scoring criteria [6] and Common Terminology Criteria for Adverse Events (version 3.0) were used to grade the late toxicities of radiotherapy.

### Statistical analysis

The chi-square test and Fisher exact test were used to compare patient characteristics between the IMRT-alone group and the IMRT/chemotherapy group. Kaplan–Meier method and the log-rank test were used to analyze OS, DFS, LRRFS, and DMFS. Multivariate Cox proportional hazards model with backward elimination was used to identify the independent predictors of different outcomes; the hazard ratios (HRs), with 95% confidence intervals (CIs), were calculated. SPSS 19.0 (IBM Corp., Armonk, NY, USA) was used for statistical analysis. All statistical tests were two-sided, and *P* ≤ 0.05 was considered statistically significant.

## Results

### Patient characteristics

Out of 215 patients assessed, 124 met the study eligibility criteria. These 124 patients included 88 men and 36 women (male–female ratio, 2.4:1), with a median age of 45 years (range 18–70 years). The 124 patients were separated into two pair-matched groups: the IMRT-alone group (*n* = 62) and the IMRT/chemotherapy group (*n* = 62). The two groups were approximate with respect to baseline characteristics (Table 1).

All patients were restaged according to the 7th edition AJCC/UICC staging system; 22 patients were reclassified as T1, 75 were reclassified as T2, and 17 as T3. In addition, 40 patients were reclassified as N0, and 84 were reclassified as N1.

### Survival outcomes

**Fig. 1:**
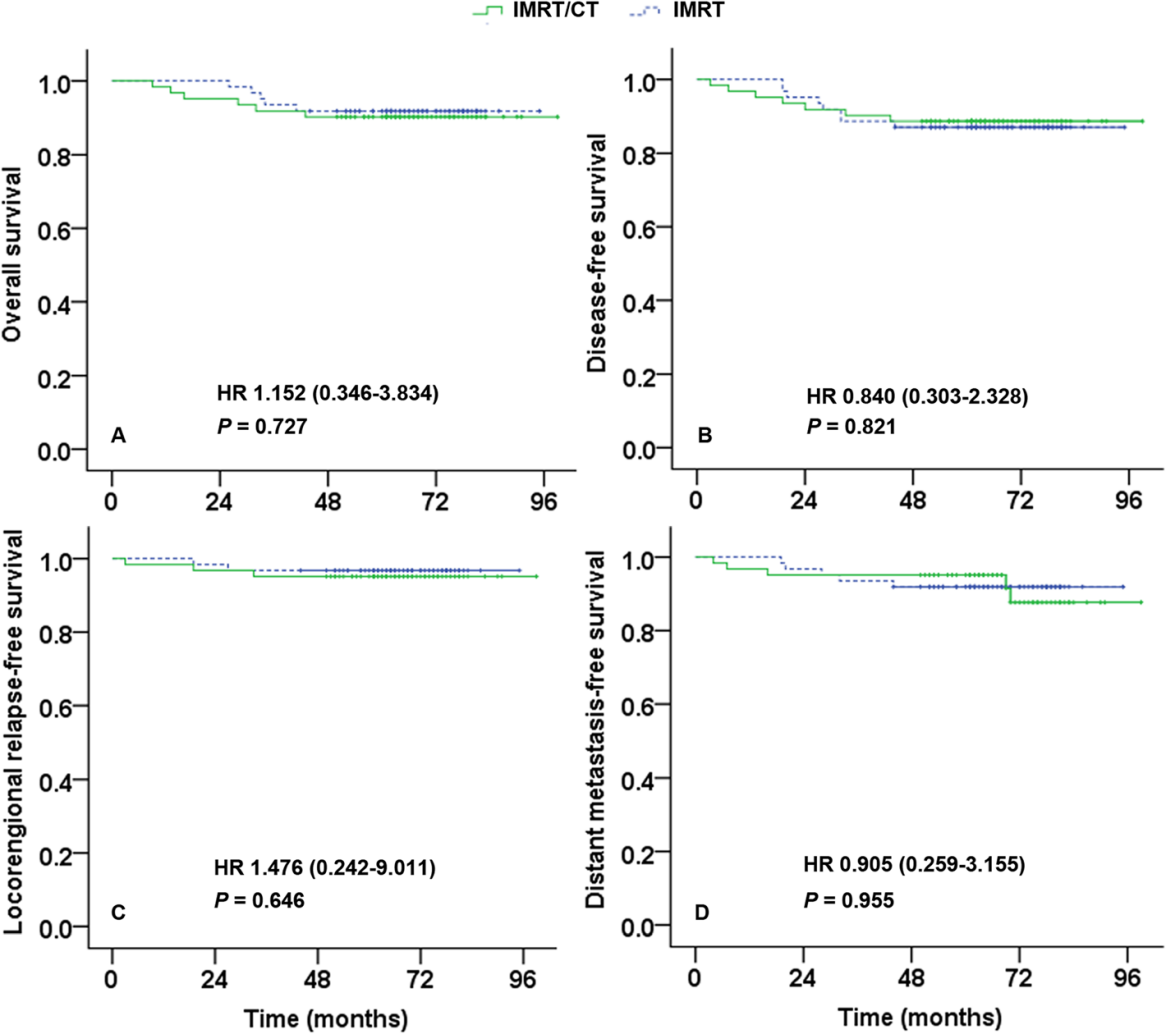
Kaplan–Meier curves for overall survival (**a**), disease-free survival (**b**), locoregional recurrence–free survival (**c**), and distant metastases–free survival (**d**) in stage II and T3N0M0 NPC patients treated with IMRT/chemotherapy and IMRT alone

Survival outcomes were approximate between the IMRT-alone group and the IMRT/chemotherapy group (Table 2): 5-year OS was 91.9% vs. 90.3%, respectively (*P* = 0.727; Fig. 1a); DFS was 87.1% vs. 88.7%, respectively (*P* = 0.821; Fig. 1b); LRFFS was 96.8% vs. 95.2%, respectively (*P* = 0.646; Fig. 1c), and DMFS was 91.9% vs. 91.5%, respectively (*P* = 0.955; Fig. 1d). Multivariate analysis (Table 3) showed that treatment (IMRT/chemotherapy vs. IMRT-alone) was not an independent prognostic factor for OS (HR = 1.152; 95% CI, 0.346–3.834; *P* = 0.818), DFS (HR = 0.840; 95% CI, 0.303–2.328; *P* = 0.738), LRFFS (HR = 1.476; 95% CI, 0.242–9.011; *P* = 0.673), or DMFS (HR = 0.905; 95% CI, 0.259–3.155; *P* = 0.875).

**Table 1:** Baseline characteristics of patients in the two groups

Characteristic	IMRT group (*n* = 62)	IMRT/chemotherapy group (*n* = 62)	*P*
**Age**			0.055
≤ 45 years	15 (24.2%)	25 (40.3%)	
> 45 years	47 (75.8%)	37 (59.7%)	
**Sex**			1.000
Male	44 (71.0%)	44 (71.0%)	
Female	18 (29.0%)	18 (29.0%)	
**Histology**			0.144
WHO type II	2 (3.2%)	6 (9.7%)	
WHO type III	60 (96.8%)	56 (90.3%)	
**T category**			0.431
T1	14 (22.6%)	18 (29.0%)	
T2	41 (66.1%)	34 (54.8%)	
T3	7 (11.3%)	10 (16.1%)	
**N category**			0.701
N0	21 (33.9%)	19 (30.6%)	
N1	41 (66.1%)	43 (69.4%)	
**TNM stage**			0.433
II	55 (88.7%)	52 (83.9%)	
III (T3N0M0)	7 (11.3%)	10 (16.1%)	

All data are n (%)

*IMRT* Intensity-modulated radiotherapy, *WHO* World Health Organization

**Table 2:** Five-year survival outcomes of patients treated with IMRT alone and IMRT plus chemotherapy

Variable	IMRT group (*n* = 62)	IMRT/chemotherapy group (*n* = 62)	HR (95% CI)	*P*
**OS (%)**			1.235 (0.377–4.048)	0.727
At 3 years	93.5%	91.9%		
At 5 years	91.9%	90.3%		
**DFS (%)**			0.890 (0.323–2.453)	0.821
At 3 years	88.7%	90.3%		
At 5 years	87.1%	88.7%		
**LRRFS (%)**			1.515 (0.253–9.066)	0.646
At 3 years	96.8%	95.2%		
At 5 years	96.8%	95.2%		
**DMFS (%)**			1.037 (0.300–3.581)	0.955
At 3 years	93.5%	95.2%		
At 5 years	91.9%	91.5%		

*IMRT* Intensity-modulated radiotherapy, *HR* Hazard ratio, *CI* Confidence interval, *OS* Overall survival, *DFS* Disease-free survival, *LRRFS* Locoregional relapse-free survival, *DMFS* Distant Metastasis-free survival

**Table 3:** Results of multivariate analysis showing the significant prognostic factors for different survival outcomes in nasopharyngeal carcinoma patients

Factor	Hazard ratio	95% CI	*P*
**Overall survival**			
Chemotherapy (IMRT vs. IMRT/CT)	1.152	0.346–3.834	0.818
Age (≤45 years vs. > 45 years)	1.307	0.347–4.930	0.692
Sex (male vs. female)	0.909	0.239–3.465	0.889
TNM stage (II vs. III [T3N0M0])	5.797	1.768–19.007	0.004
**Disease-free survival**			
Chemotherapy (IMRT vs. IMRT/CT)	0.840	0.303–2.328	0.738
Age (≤45 years vs. > 45 years)	1.049	0.351–3.135	0.932
Sex (male vs. female)	0.590	0.166–2.092	0.414
TNM stage (II vs. III [T3N0M0])	4.990	1.772–14.047	0.002
Locoregional relapse-free survival			
Chemotherapy (IMRT vs. IMRT/CT)	1.476	0.242–9.011	0.673
Age (≤45 years vs. > 45 years)	0.832	0.136–5.080	0.842
Sex (male vs. female)	1.015	0.212–3.457	0.973
TNM stage (II vs. III [T3N0M0])	1.575	0.176–14.099	0.685
**Distant metastasis-free survival**			
Chemotherapy (IMRT vs. IMRT/CT)	0.905	0.259–3.155	0.875
Age (≤45 years vs. > 45 years)	1.075	0.270–4.271	0.919
Sex (male vs. female)	0.950	0.241–3.736	0.941
TNM stage (II vs. III [T3N0M0])	4.705	1.322–16.751	0.017

*IMRT* Intensity-modulated radiotherapy, *CI* Confidence interval, *CT* Chemotherapy

**Table 4:** Treatment-related toxicities in the two groups

Toxicity	IMRT arm (*n* = 62)		IMRT/CT arm (*n* = 62)		*P*
	Grade 3	Grade 4	Grade 3	Grade 4	
Skin reaction (radiation-related)	4	0	10	0	0.089
Mucositis (radiation-related)	5	0	15	0	0.004
Vomiting /Nausea	3	0	22	0	< 0.001
Leukopenia/neutropenia	1	0	8	0	0.015
Thrombocytopenia	0	0	2	0	0.496
Anemia	0	0	2	0	0.496
Dry mouth	2	0	2	0	1.000
Nephrotoxicity	0	0	0	0	–
Hepatoxicity	0	0	0	0	–

*IMRT* Intensity-modulated radiotherapy

**Table 5:** Analysis of survival outcomes in different subgroups of the IMRT group versus the IMRT/chemo-therapy group

Factor	OS	*P*	DFS	*P*	LRRFS	*P*	DMFS	*P*
**Age**								
≤ 45 years	86.7% vs. 96.0%	0.282	80.0% vs. 92.0%	0.238	93.3% vs. 96.0%	0.696	86.7% vs. 96.0%	0.299
> 45 years	93.6% vs. 86.5%	0.257	89.4% vs. 86.5%	0.620	97.9% vs. 94.6%	0.415	93.6% vs. 94.6%	0.369
**Sex**								
Male	90.9% vs. 90.9%.	0.980	84.1% vs. 88.6%	0.581	95.5% vs. 93.2%	0.643	90.9% vs. 95.5%	0.708
Female	94.4% vs. 88.9%	0.553	94.4% vs. 88.9%	0.553	100% vs .100%	1.000	94.4% vs. 94.4%	0.504
**T category**								
T1	100% vs. 94.4%	0.378	100% vs. 94.4%	0.378	100% vs. 94.4%	0.378	100% vs. 100%	1.000
T2	92.7% vs. 94.1%	0.825	87.8% vs. 91.2%	0.639	97.6% vs. 94.1%	0.452	90.2% vs. 94.1%	0.571
T3	71.4% vs 70.0%	0.907	51.1% vs. 70.0%	0.791	100% vs. 100%	1.000	71.4% vs. 90.0%	0.362
**N category**								
N0	90.5% vs. 84.2%	0.539	85.7% vs. 84.2%	0.817	95.2% vs. 100%	0.342	95.2% vs. 94.7%	0.215
N1	92.7% vs. 93.0%	0.964	87.8% vs. 90.7%	0.678	97.6% vs. 93.0%	0.331	90.2% vs. 95.3%	0.389
**TNM stage**								
II	94.5% vs. 94.2%	0.935	90.9% vs.92.3%	0.802	98.2% vs.94.2%	0.283	92.7% vs. 96.2%	0.462
III (T3N0M0)	71.4% vs. 70.0%	0.907	57.1% vs.70.0%	0.801	85.7% vs.100%	0.232	85.7% vs. 90.0%	0.648

*OS* Overall survival, *DFS* Disease-free survival, *LRRFS* Locoregional relapse–free survival, *DMFS* Distant metastasis–free survival

### Treatment toxicities and compliance

All patients in both treatment arms completed the prescribed dose of IMRT. However, not all patients completed chemotherapy; the reasons for withdrawal of cisplatin included refusal by the patient, severe mucositis, and prolonged severe neutropenia. The leading grade 3 acute toxic effects during treatment were hematologic and gastrointestinal reactions (Table 4). No grade 4 or 5 toxicity (death) occurred during treatment. The incidence of grade 3 acute toxic effects was significantly higher in the IMRT/chemotherapy group than in the IMRT-alone group: the incidence of grade 3 hematologic toxicity (leukopenia/neutropenia) was 8% vs. 1%, respectively (*P* < 0.015); the incidence of grade 3 gastrointestinal toxicity (nausea/vomiting) was 22% vs. 3%, respectively (*P* < 0.001); and the incidence of grade 3 mucositis was 15% vs. 5%, respectively (*P* = 0.004). No patient had grade 4 mucositis.

### Subgroup analysis

Subgroup analysis was performed by age (≤45, > 45 years), sex (male, female), T category (T1, T2, T3), N category (N0, N1), and AJCC stage (stage II, III [T3N0M0]). No significant differences were found between the different strata in each subgroup (Table 5).

## Discussion

This study directs to set side by side the efficacy and safety of IMRT plus chemotherapy versus IMRT alone in intermediate-risk NPC patients. We found approximate survival outcomes (5-year OS, DFS, LRFFS and DMFS) with IMRT alone and IMRT plus chemotherapy. Treatment-related toxicities were significantly more in the group treated with IMRT plus chemotherapy.

Nowadays, satisfactory disease control is relatively easily achieved with multimodality treatment in a variety of malignancies; however, the focus is to individualize treatment to achieve the best possible results in each patient. There are still some differing opinions on whether the benefit gained with radiotherapy in stage II and T3N0M0 NPC could be enhanced by the addition of chemotherapy. Guo et al. reported that the addition of chemotherapy could improve LRRFS (HR = 0.263, 95% CI: 0.083–0.839; *P* = 0.024), especially for T1N1 patients (HR = 0.209, 95% CI: 0.046–0.954; *P* = 0.043) [11]. However, our pair-matched analysis of 124 stage II and T3N0M0 NPC patients showed no significant survival benefit with the use of IMRT plus chemotherapy. Our results are in line with a retrospective study by Xu et al. that demonstrated similar OS, LRRFS, and DMFS in stage II NPC patients treated with IMRT alone and with IMRT plus chemotherapy. Patients with T3N0M0 NPC represent a group at special risk for distant metastasis, but our study found almost similar 5-year OS in patients treated with IMRT plus chemotherapy and with IMRT alone. Multivariate analyses showed that treatment regimen (IMRT/chemotherapy vs. IMRT-alone) was not an independent prognostic factor for OS in these patients. A meta-analysis by Cheng et al. also found similar OS, LRRFS, and DMFS in stage III (T3N0M0) NPC patients treated with CCRT and with IMRT alone [12]. In subgroup analysis, The IMRT plus chemotherapy showed longer DFS compared to the IMRT alone in stage III (T3N0M0) NPC patients, The possible reasons may be due to the small number of patients in T3N0M0 subgroup. The benefits of adding chemotherapy to T3N0M0 needs to be confirmed by prospective studies.

In the present study, the overall incidence of grade 3 acute toxic effects was higher in the IMRT/chemotherapy group than in the IMRT-only group; significantly higher incidence was seen of grade 3 leukopenia/neutropenia, nausea/vomiting, and mucositis. Some earlier studies have reported similar findings [7, 13]. We separated the patients into different subgroups according to various baseline factors. No significant difference was seen between the different strata in each subgroup. Therefore, assessment of patients with precise population stratification may reduce the benefits of CCRT to a non-significant effect, which could apply equally to other diseases.

We offer two possible explanations for the similarity in survival outcomes between the IMRT/chemotherapy and the IMRT-alone groups in this study. First, IMRT provides better local tumor control than 2D-RT [14, 15] and so the potential gains achieved with the addition of chemotherapy may not as obvious [16]. Second, the high frequency of severe adverse reactions in patients treated with IMRT/chemotherapy may have masked any survival benefit in this group [17]. Lan et al. have also found that addition of chemotherapy to IMRT does not significantly improve OS; additionally, the authors reported that the higher the incidence of grade 3–4 acute toxicities (especially hematological events such as leucopenia and neutropenia [13]) increases the possibility of discontinuation of treatment. Thus, it seems that IMRT alone may be more suitable than CCRT for patients with stage II and T3N0M0 NPC.

Some limitations of this study should be kept in mind when interpreting the results. First, this is a retrospective study, and some bias is inevitable; for example, patients received different chemotherapy regimens, and this may have influenced the results. Second, the sample size was relatively small.

## Conclusion

In intermediate-risk (stage II and T_3_N_0_M_0_) NPC, IMRT provides survival outcome comparable to that with IMRT plus chemotherapy. Moreover, grade 3 acute toxicities are fewer with IMRT alone than with IMRT plus chemotherapy. Well-designed large randomized clinical trials comparing CCRT with IMRT alone are needed to confirm our findings and to help formulate individualized therapies for stage II and T3N0M0 NPC patients.

## Data Availability

All data generated or analysed during this study are included in this published article.

## Abbreviations

2D-RT: Two-dimensional radiotherapy
3DCRT: Three-dimensional conformal radiotherapy
CCRT: Concurrent chemoradiotherapy
CECT: Contrast-enhanced computed tomography
CI: Confidence interval
CTV: Clinical target volume
DFS: Distant failure–free survival
DMFS: Distant metastasis–free survival
GTVnd: Metastatic cervical lymph nodes
GTVnx: Gross tumor volume
HR: Hazard ratio
IMRT: Intensity-modulated radiotherapy
LRRFS: Locoregional relapse–free survival
MRI: Magnetic resonance imaging
NPC: Nasopharyngeal carcinoma
OS: Overall survival
PTV: Planning target volume
RTOG: Radiation Therapy Oncology Group
UICC/AJCC: International Union against Cancer/American Joint Committee on Cancer
WHO: World Health Organization
